# Welcome to Wonderland: The Influence of the Size and Shape of a Virtual Hand On the Perceived Size and Shape of Virtual Objects

**DOI:** 10.1371/journal.pone.0068594

**Published:** 2013-07-11

**Authors:** Sally A. Linkenauger, Markus Leyrer, Heinrich H. Bülthoff, Betty J. Mohler

**Affiliations:** 1 Max Planck Institute for Biological Cybernetics, Tübingen, Germany; 2 Department of Brain and Cognitive Engineering, Korea University, Seoul, South Korea; Colorado State Univeresity, United States of America

## Abstract

The notion of body-based scaling suggests that our body and its action capabilities are used to scale the spatial layout of the environment. Here we present four studies supporting this perspective by showing that the hand acts as a metric which individuals use to scale the apparent sizes of objects in the environment. However to test this, one must be able to manipulate the size and/or dimensions of the perceiver’s hand which is difficult in the real world due to impliability of hand dimensions. To overcome this limitation, we used virtual reality to manipulate dimensions of participants’ fully-tracked, virtual hands to investigate its influence on the perceived size and shape of virtual objects. In a series of experiments, using several measures, we show that individuals’ estimations of the sizes of virtual objects differ depending on the size of their virtual hand in the direction consistent with the body-based scaling hypothesis. Additionally, we found that these effects were specific to participants’ virtual hands rather than another avatar’s hands or a salient familiar-sized object. While these studies provide support for a body-based approach to the scaling of the spatial layout, they also demonstrate the influence of virtual bodies on perception of virtual environments.

## Introduction

The influence of perceivers’ bodies on perception was originally introduced by Gibson [1], who stressed that individuals do not perceive the environment, but rather they perceive the *relationship* between their body and the environment. More specifically, he asserts, “Children learn to see sizes in terms of prehension: they see the span of their grasp and the diameter of a ball at the same time. Long before the child can discriminate one inch, or two, or three, he can see the fit of the object to the pincer-like action of the opposable thumb. The child learns his scale of sizes as commensurate with his body, not with a measuring stick (pgs. 234-235).” For the most part, our body size determines the range of potential actions we can perform within the environment, and thereby, defines the interactive value of the objects of which our environment is composed.

Consider a popular example from classic literature. In *Alice’s Adventures in Wonderland* by Lewis Carroll [2], Alice, the protagonist, shrinks to the size of a child’s doll after drinking a mysterious liquid out of a bottle labeled “Drink Me”. In an attempt to remedy this frightful situation, Alice eats a bite of a cake labeled “Eat Me” after which she grows to a height of nine feet tall. These changes provide a striking illustration of how the size of one’s body can affect the ability to interact with objects in the surrounding environment. To big Alice, the objects surrounding her have become miniature (the white rabbit could fit in the palm of her hand); to small Alice, the objects surrounding her have become massive (the white rabbit looms over her). As a result, big Alice can pick up much larger objects and reach much farther than little Alice. Drawing from Gibson’s [1] account, one could hypothesize that although in the same physical surroundings, big Alice and little Alice perceive two distinctly different spatial environments, because the interactive value of the objects of which the environment is composed drastically differs following a change in the relationship between Alice’s size and the size of the environment.

From this reasoning, a new approach to the perception of spatial layout, hereafter referred to as body-based perceptual scaling, has provided a potential explanation as to how the environment is perceived relative to the size of the individual [3]. Visual information that specifies extents comes to the eye in the form of visual angles and changes in visual angles and in order to perceive an extent that angular information needs to be scaled to a metric that is appropriate for extent. Body-based perceptual scaling approaches contend that the perceptual system uses the dimensions of the body and its action capabilities as “perceptual rulers” to which optical information is rescaled to a metric which is appropriate for determining the perceived extents ( [3] for a review). As a result, extents are perceived as a proportion of the action capability to which the extent is relevant. For example, little Alice would perceive the “Drink Me” bottle as being a proportion of the maximum sized object that she can grasp, and for our purposes, let us assume that the bottle is about 50% of her maximum grip aperture. However, because big Alice has much larger hands, she can grasp much larger objects, and the bottle is now only 5% of her maximum grip aperture. Because the object measures as smaller on big Alice’s larger “perceptual ruler”, she perceives an object of the same physical size as being smaller than normal Alice. If this is the case, then the perception of sizes and distances is scaled using our body and the action capabilities of our body as a metrics for perceiving the spatial properties of the surrounding visual world.

The body-based perceptual scaling approach attests that many of the perceptual metrics used to scale perceived extents are derived from the functional morphology of the body. Here, we refer to functional morphology as the possible actions that can be performed as a result of the form, structure, and size of the body (other perceptual metrics are derived from physiological and skill based metrics; however, we are not addressing this here). Therefore, this term not only specifies what type of actions can be performed, but additionally, the extent over which the action can be performed. Referring to the previous example, both big and little Alice have hands that allow for grasping; however, due to the differences in their hand sizes, big Alice can grasp larger objects than little Alice. The type of action defines the relevant perceptual ruler, i.e. the hand when grasping; however, the size of the hand defines the size of the perceptual ruler. In this manuscript, we are primarily focusing on the role of hand size as a perceptual ruler for object size. However, there is ample empirical support that several other manipulations of our functional morphology act as perceptual scaling metrics, such as arm’s reach [4–6], eye-height [7–9], shoulder width [10], jumping ability [11], and overall body size [12].

The studies presented in this paper attempt to answer the question whether graspable objects are scaled to the apparent size/grasping ability of the hand. Previous research has found support that the visual perception of hand size can influence the perception of object size. For example, after magnifying the hand by placing it into a magnification box, individuals perceive the sizes of non-magnified objects as being visually smaller [13]. Additionally, right-handed individuals perceive their right hand as larger and capable of grasping larger objects than with their left hand [14]. Concordantly, right-handed individuals perceive graspable objects as smaller when intending to grasp them with their right hand than when intending to grasp them with their left [13]. Although these studies provide some evidence for the body-based perceptual scaling approach, these experiments also highlight how limited researchers are in their ability to manipulate the perceived dimensions of the hand. Magnification and minification of only the hand in one’s visual environment is possible using a magnifying or minifying sheet over only one’s hand. The same applies to distorting the dimensions of the hand using prism goggles. Even in cases where this could be achieved, we are still limited to changes afforded by the optical properties of these lenses. The problem of assessing the influence of the body on perception comes down to the need to have precise control over the changes in the dimensions of perceivers’ bodies.

With recent advances in technology, more control over the perception of the dimensions of the body can be achieved using virtual reality. Several experiments have presented participants with immersive, full cue virtual environments using state-of-the-art head mounted displays (HMDs). By adding motion tracking, researchers can provide participants with “virtual bodies” by mapping body movements to self-representing avatars in the virtual world. Participants see these self-representing avatars from a first-person perspective, and the movements of the participant are mapped onto the movements of the self-representing avatar in real time. The result is the visual experience of being in an immersive, realistic virtual environment with a fully-animated body.

Several studies have shown that perceptual-motor synchrony is sufficient to produce embodiment effects [15,16], which means that the participants are seeing their virtual bodies as representations of themselves in the new environment. Furthermore, a recent study demonstrates that people also display physiological responses such as deceleration in heart rate in response to threats to their avatar [17]. Therefore, these results provide support that self-representing avatars are treated as a convincing representation of the individual’s own body in the virtual environment.

As a consequence, using virtual reality technology, one can easily manipulate the perception of individuals’ bodies by modifying aspects of the self-representing avatar’s body. Even when the virtual body drastically deviates from the physical body, by maintaining the perceptual-motor fidelity between individuals and their avatars, individuals still experience the avatar as a representation of themselves as evidenced by their reactions to changes in the virtual body as if they were changes in their own body [15,18]. For example, even after extending the length of the virtual arm to be several meters long, individuals’ show intense physiological responses in the event that the virtual limb appears to be in threat [19]. Virtual bodies that have shortened limbs can create the experience that their perceiver’s own limb is shorter [20]. Hence, using virtual environments and fully animated, self-representing avatars allows us to recreate experiences similar to those Alice experienced in Wonderland.

Drawing from these findings, we can control perceived dimensions of the body more precisely by providing individuals with virtual bodies in virtual environments. If the body is used to scale the perceived environment, then manipulating the virtual body should influence the perception of the virtual environment. By using virtual reality, we can employ larger manipulations than previously employed in previous experiments and in turn, investigate the size of the effects. Using these methods, we can also explore whether less transparent measures change in size perception (i.e. shape perception) occur as a result of body-based scaling. These studies also have strong implications in applied domains as they investigate the level of importance of the size and shape of the virtual body on the perception of the other elements within the virtual environments. In a set of studies, we investigated the influence of the dimensions of a self-representing virtual hand on the perception of size and shape of objects within a virtual environment. If the perceptual system uses the virtual body as a metric to scale the sizes of perceived objects, then changes in the dimension of the avatar’s hand should affect the perception of the size and shape of objects within the virtual environment.

### Experiment 1: Virtual Hand Size and Size Perception

In this experiment, we investigated the impact of the size of the individual’s virtual hand on the perceived sizes of objects within the virtual environment. Participants experienced different sized virtual hands which were fully animated in real time while making judgments of the sizes of spheres. If participants scale the apparent sizes of objects to their virtual hands, then objects should appear smaller as their virtual hand size increases.

## Methods

### Participants

Twelve (4 female) individuals participated in this experiment. Participants were recruited from the university community around Tübingen, Germany and were compensated for their time at a rate of eight € per hour. All participants started the experiment by completing a written consent form, which along with this study was approved by the ethical committee of the University of Tübingen, Germany for this study. All participants had normal or corrected to normal vision. All participants in this and the subsequent studies provided informed consent.

### Stimuli and Apparatus

**Figure 1:**
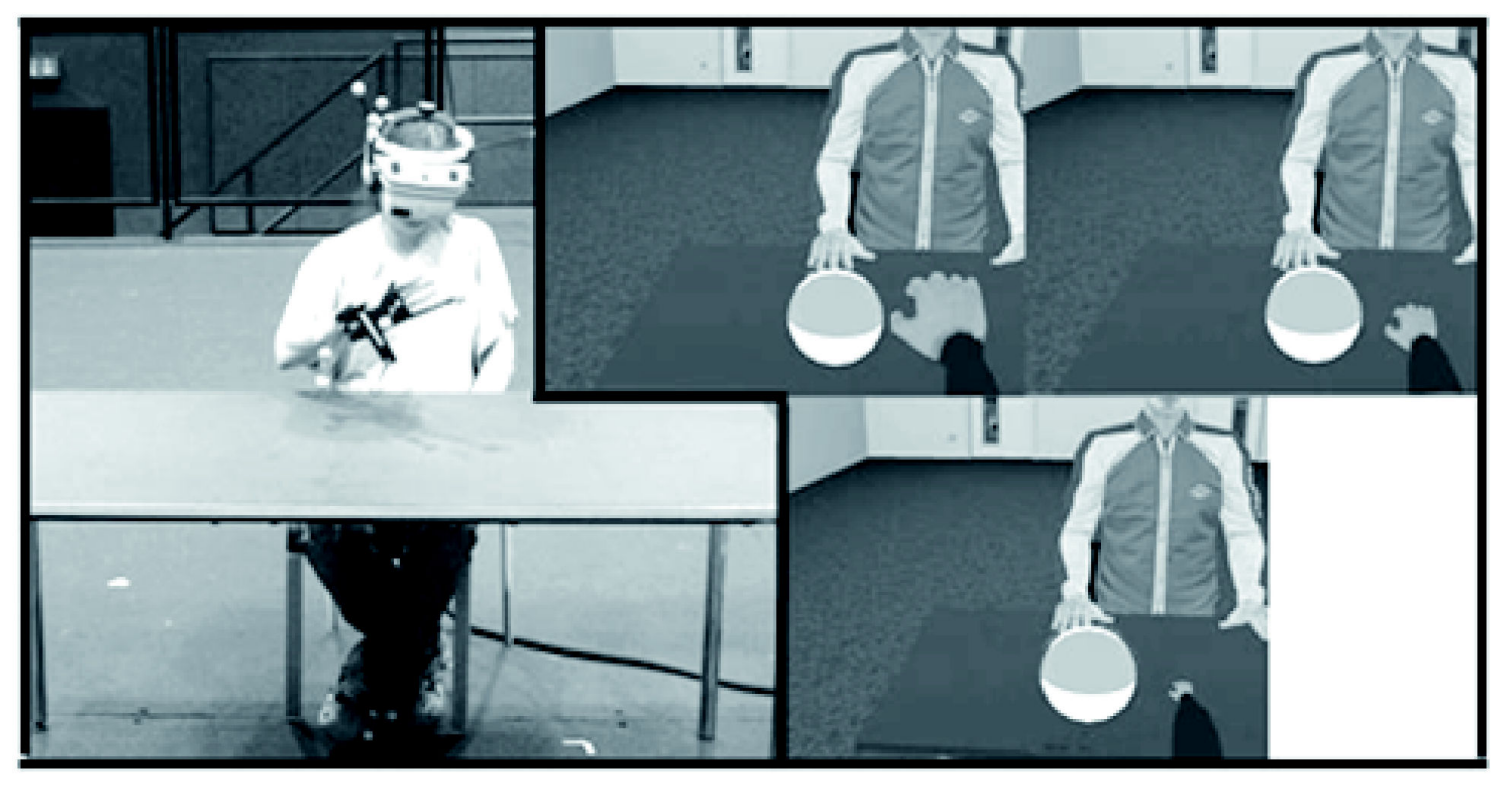
Left: Participant in experimental set-up; Right: View of participant for the three different hand size conditions (large, medium, small).

The experiment was conducted in a large, fully tracked walking space (12m x 12m). A table (72cm high, 150cm in length, and 75cm in width) and chair (seat height of 44cm) were placed in the center of the walking space. The position of participants’ hands and heads were tracked using an optical tracking system (16 Vicon MX13 cameras) through the monitoring of reﬂective markers. Each Vicon camera has a resolution of 1280x1024 and the tracking system has an average end-to-end latency of 40.8 ms (end-to-end latency refers to the time needed from moving the head, tracking the movement and updating the virtual environment accordingly) measured with the method proposed by di Luca [21]. To effectively track the hand, participants wore a small light weight (120 g) flat metal disk strapped to the top of their right hand on which four reflective markers were attached. Participants wore the nVisor SX60 head-mounted display (HMD) which displayed a stereoscopic image of the virtual world with a resolution of 1280x1024 pixels per eye at a frame rate of 60 Hz and a FOV of 60 degrees diagonally. The HMD also had reflective markers attached, which allowed its position and orientation to be tracked in real-time (see Figure 1).

For the virtual environment, we used a 3D model of a real room and its contents. The materials in the scene were made to be as realistic as possible, and tables, chairs, book cases, a kitchenette, an air-conditioner, doors, and posters were modeled and added into the virtual office to provide familiar size cues. The 3D room was modeled in Autodesk 3ds Max 2009 [22]. The textures used in the scene were extracted from photos of the real room. In the middle of the virtual environment, we placed a virtual table and chair whose dimensions and positions were the same as the real table and chair in the tracking space. Across the table from the chair, a male avatar was seated with his hands flat on the table to provide biological familiar size cues (see Figure 1). Twelve different sized virtual balls were created as stimuli ranging from 4 to 21 cm in diameter. They were textured with a white and gray checkered material. The image displayed on the HMD was updated with the position of the tracked head and hand so that it was consistent with the participant’s head and hand movements. The movement of the participant’s tracked hand was mapped onto the virtual arm and hand, so that the virtual hand appeared to move as the participant’s hand moved in the same location as the physical hand. Inverse kinematics were used in a manner that ensured that the virtual location of the hand was the same location as the physical hand location, while the exact location of the virtual and physical elbow were not guaranteed to match.

### Procedure

Participants were asked to sit in the chair facing the center of the table in the tracking space. The metal disk was strapped to their right hand and participants donned the HMD. Participants were told that they were going to be presented with several differently sized balls, one at a time, and they were to estimate how big the ball appeared. Participants were instructed to place their hand on the table next to the position that the ball would appear. The ball appeared in the middle of the table in-between the participant and the avatar sitting across from the participant. They estimated size using a verbal scale, with 0 being the size of a bean and 10 being the size of a basketball. Participants were able to respond above 10 or below 0 for objects in which they thought were outside of the range of the scale. Participants were also instructed to be as accurate as possible and to employ fractional units if necessary. Every participant reported being at ease with using the verbal scale. The stimulus remained in front of the participant until they provided a verbal estimate they were satisfied with. Following their response, a black screen occluded the participants’ view of the virtual environment. After 500ms, a new trial began as the black screen was dropped to show the virtual environment with the next stimulus. Participants completed 3 blocks of estimating the size of all 12 balls in each block for a total of 36 trials. Ball presentation was randomized within each block. Each block differed by the size of the virtual hand; the sizes of participants’ virtual hands were small (Width: 3.5 cm, half), medium (Width: 7 cm), or large (Width: 14 cm; doubled). Hand size block order was counterbalanced across participants.

## Results

**Figure 2:**
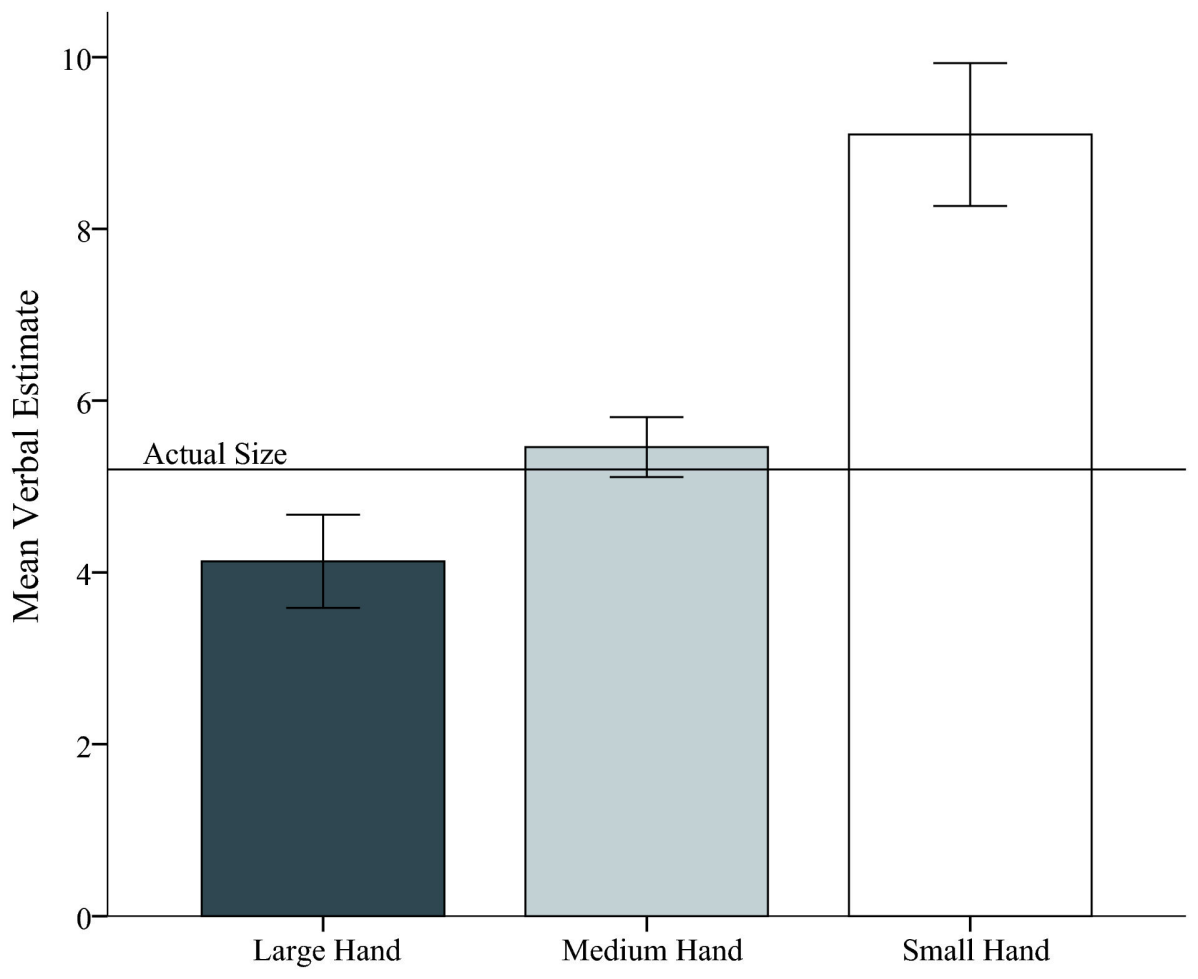
Mean verbal estimate of spheres for the three conditions of hand size (large, medium and small). Error bars represent 1 standard error and are calculated on the basis of within-participant error with the method provided by Loftus and Masson [37]. The line labeled “Actual Size” indicates the mean of actual physical size of the stimuli with respect to the verbal scale.

**Figure 3:**
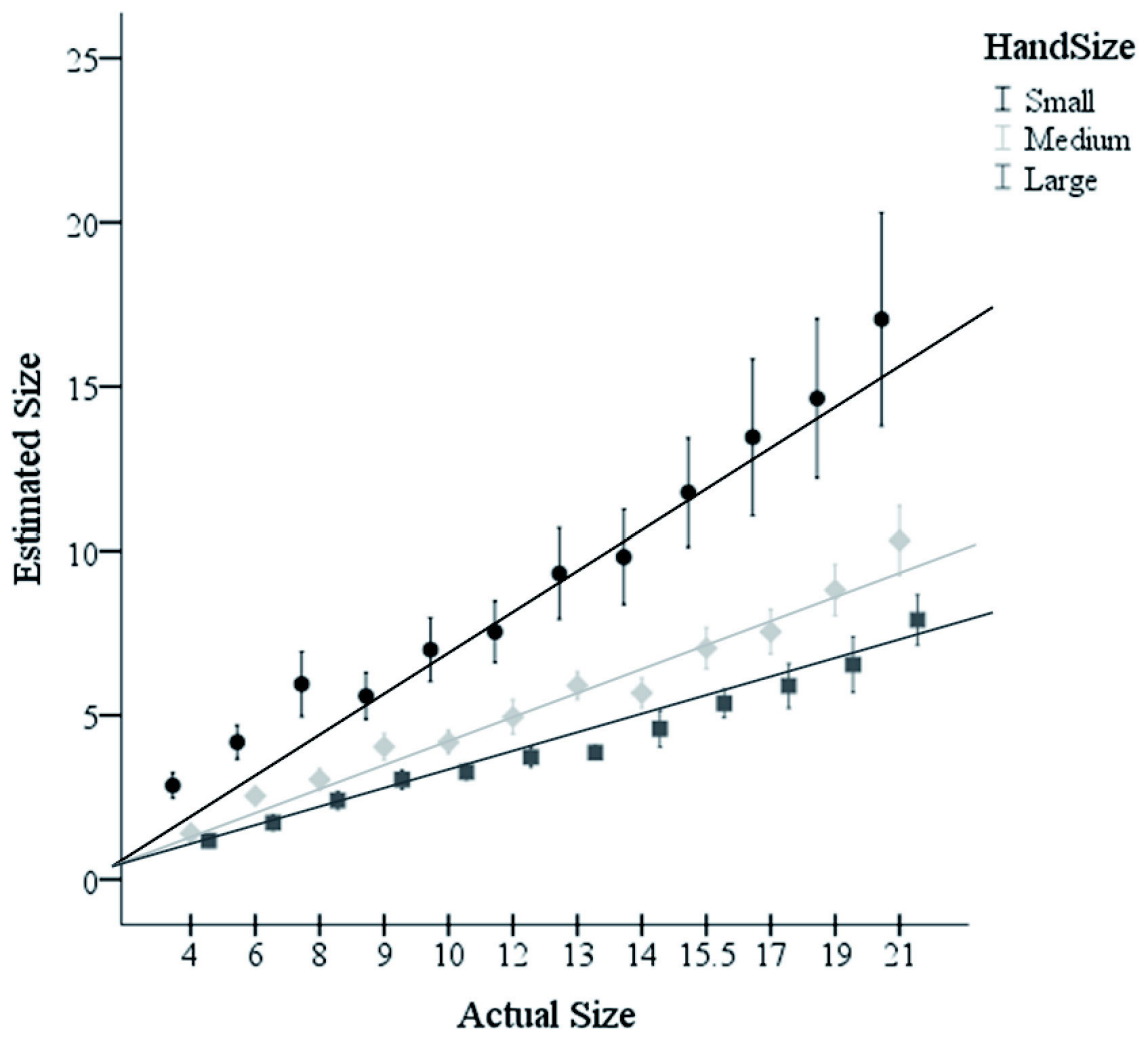
Linear trends of the verbal estimates across different sized objects in the three hand size conditions. Error bars represent 1 standard error.

The data from one participant was removed from the analysis, because their estimates were 3 SD above the mean. A repeated measures analysis of variance (ANOVA) was conducted with hand size (large, normal, or small) and ball size (12 sizes) as independent variables and size estimate as the dependent variable, see Figure 2. As hypothesized, hand size significantly influenced size estimates, *F*(2,20) = 11.96, *p*< .01, *ŋ*_*p*_^2^ = .55. Fisher’s LSD post-hoc comparisons show that the estimates in the large hand condition, *M*= 4.12, *SE* = .31, were significantly smaller than in the medium hand, *M* = 5.45, *SE* = .43, *p* < .01, and the small hand conditions, *M* = 9.10, *SE* = 1.30, *p* < .01. The medium hand condition was also significantly different from the small hand condition, *p* < .01. There was also a significant effect of object size with larger objects being estimated as larger than smaller objects, *F*(11,110) = 46.85, *p* < .01, *ŋ*_*p*_^2^ = .82. Additionally, there was an interaction between hand size and object size, *F*(22,220) = 3.96, *p* < .01, *ŋ*_*p*_^2^ = .28, with the differences between the hand size conditions increasing as a function of increases in object size, see Figure 3. This interaction is in support of the body-based perceptual scaling hypothesis, because increases as a function of size are indicative of differences in a scaling metric rather than a consistent bias. Put simply, if these results were due to a response bias, then we would expect that individuals would be consistent in their changes in response regardless of stimulus size. Therefore, response bias would be evident in the data as an intercept difference and no slope difference between the different conditions when regression lines are plotted with estimated size across the different physically sized stimuli. However, if the data is more in line with the body based scaling account and different sized “perceptual rulers” are used in the different conditions, then the difference between the two conditions should increase as a function of physical size. This hypothesis would result in slope differences between the two conditions but no intercept difference.

In order to look at this more closely, slopes and intercepts of straight lines fit using least squares regression for each participant in each condition were calculated. Two repeated measures ANOVAs were conducted with hand size as the independent variable; however, one had slope as the dependent measure and the other had intercept as the dependent factor. As predicted, the slopes were significantly different across hand size conditions, *F*(2,20) = 5.49, *p* = .01, *ŋ*_*p*_^2^ = .35. Intercept did not differ across hand conditions, *p* = .81, as would be predicted by changes in the scaling metric.

These results suggest that changes in the size of the virtual body can influence perceived size. Similarly, the results are consistent with the account that individuals use the size of their virtual hand as a perceptual metric to scale the apparent size of the virtual objects.

### Experiment 2: Avatar Hand Size and Size Perception

Although the previous experiment suggests that size perception can be influenced by one’s own body, there is still the possibility that the results in Experiment 1 were due to size contrast effects rather than body-based perceptual rescaling. One example is the popular Ebbinghaus illusion in which objects appear smaller or larger depending on the sizes of the objects around them [23]. Therefore, it is possible these results may not be due to body-based perceptual scaling, but rather the size contrast between the size of the hand and the target object. Additionally, the results from Experiment 1 do not rule out the possibility that it was not one’s own body that is special, but parts of any person’s body, i.e. even another’s body can influence size perception. As a result, we conducted Experiment 2 where we used the same method as in Experiment 1, except instead of manipulating the participant’s virtual hand size, we manipulated the hand size of the virtual character sitting across the table from the participant.

### Methods

#### Participants

Ten (3 female) participants were recruited from the university community around Tübingen, Germany participated in the experiment. All participants started the experiment by completing a written consent form, which along with this study was approved by the ethical committee of the University of Tübingen, Germany for this study. Participants were compensated for their time at a rate of eight € per hour.

#### Stimuli & Apparatus

The experiment was conducted in a large, fully tracked space (4m x 6m). The same table and chair from Experiment 1 were placed in the center of the walking space. The table was covered with a black table cloth to decrease interference in the trackers. The position of participants’ hand and head were tracked using an optical tracking system (4 Vicon MX13 cameras, same specifications as in Experiment 1). Participants wore the same tracking apparatus on their hand as in Experiment 1. Participants wore the Kaiser SR80 Proview HMD which displayed a stereoscopic image of the virtual world with a resolution of 1280x1024 pixels, a frame rate of 60 Hz, and a FOV of 80 degrees diagonally. We used this particular HMD, because it has a larger field of view than the HMD used in Experiment 1, which allowed participants to better see the virtual character as well as the spheres. The virtual room model was the same as was used in Experiment 1. The stimuli were the same 12 balls as used in Experiment 1.

#### Procedure

**Figure 4:**
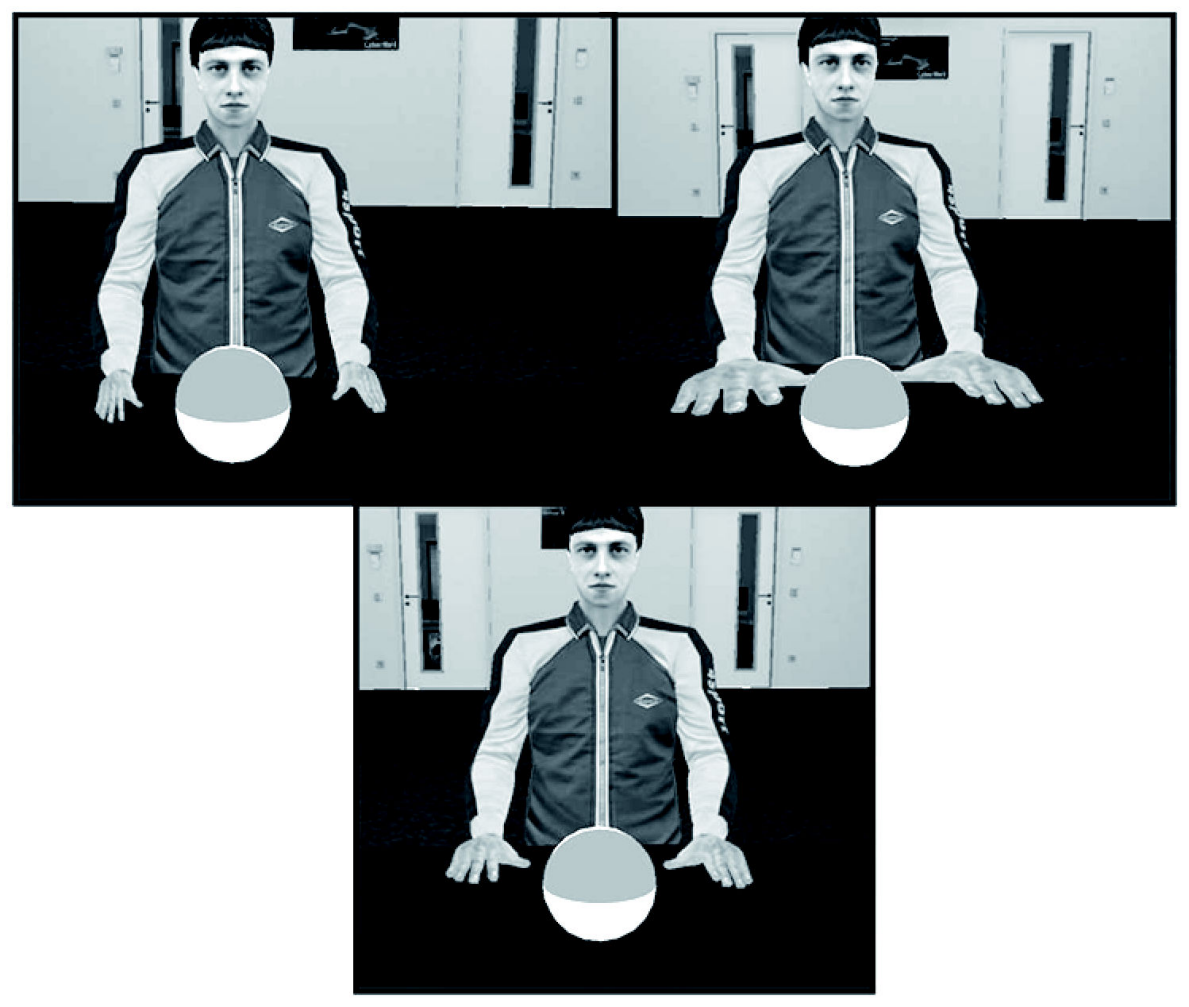
Experimental setup for Experiment 2 including the views of the participant of the virtual male character and his three different hand sizes for the different conditions.

The procedure was the same as in Experiment 1; however, instead of manipulating the size of the participants’ virtual hand, the sizes of the static male virtual character’s hands were manipulated across blocks. The change in hand size of the virtual character corresponded to the change of size of the virtual self-hand in Experiment 1 (see Figure 4). The sizes of these virtual hands were small (W: 5 cm), medium (W: 10 cm), or large (W: 14 cm). Participants had a self-animated hand as in Experiment 1, except in all conditions, it was the medium sized hand. The balls were moved to be positioned between the static male avatar’s right and left hands.

## Results

**Figure 5:**
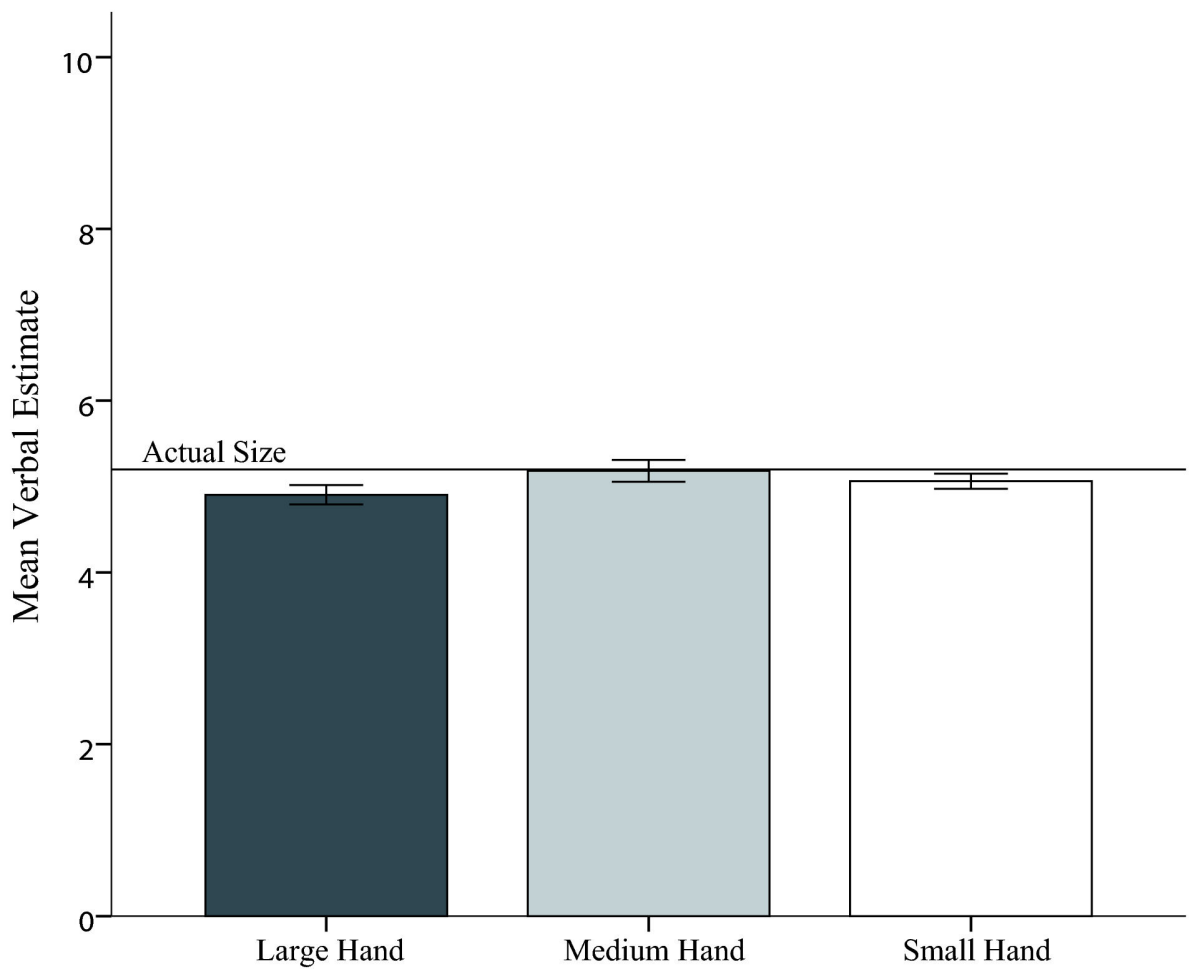
Size estimates for participants across the different avatar hand size conditions. Error bars represent 1 standard error and are calculated on the basis of within-participant error with the method provided by Loftus and Masson [37]. The line labeled “Actual Size” indicates the mean of actual physical size of the stimuli with respect to the verbal scale.

Size estimates were analyzed using a repeated measures ANOVA with actual ball size and hand size as within-subjects variables and size estimate as the dependent variable. Actual ball size was significant with individuals giving larger estimates for larger balls, F(11,99)= 151.77, *p*< .01, *ŋ*_*p*_^2^ = .94. Hand condition was not significant, *F*(2,18) = 1.08, *p*=.36, with individuals providing similar estimates in the large, *M*= 4.91*, SE*=.30, medium, *M*=5.19*, SE*=.38, and small hand, *M*=5.07*, SE*=.32, conditions, see Figure 5.

These results support the notion that the results in Experiment 1 were not due to size contrast, because even in the presence of the differently sized avatar hands, the perceived sizes of the target objects were not influenced. Similarly, these results suggest that body-based perceptual scaling is likely specific to one’s own body rather than another’s static body.

### Experiment 3: Familiar Sized Object and Size Perception

As we argue that the body has a privileged role for scaling our environment, we conducted another control study. While the results of Experiment 2 suggest that the difference in Experiment 1 is not due to size-contrast effects, it does not rule out the possibility that the virtual hand of the participant serves as the most familiar size cue in the virtual environment. Because the virtual hand of the participant is a familiar depth cue whose location very close to the spheres to be estimated, we conducted Experiment 3, where we introduced another familiar object (here a virtual fountain pen) which was placed directly next to the spheres. If the perceptual system uses the body as a metric to scale perceived object size, then changes in the dimension virtual pen should not affect the perception of the size and shape of objects within the virtual environment.

### Methods

#### Participants

Twelve (5 female) individuals participated in this experiment. Participants were recruited from the university community around Tübingen, Germany and were compensated for their time at a rate of eight € per hour. All participants started the experiment by completing a written consent form, which along with this study was approved by the ethical committee of the University of Tübingen, Germany for this study.

#### Stimuli & Apparatus

The experiment was conducted in a large, fully tracked walking space (4m x 6m). The same table and chair from Experiment 1 were placed in the center of the walking space. The table was covered with a black table cloth to decrease interference with the tracking technology. The position of participants’ hand and head were tracked using an optical tracking system (4 Vicon MX13 cameras, same as in Experiment 2). Participants wore the same tracking apparatus on their hand as in Experiment 1. Participants wore the nVisor SX60 head-mounted display (HMD); the same HMD as used in Experiment 1.

#### Procedure

**Figure 6:**
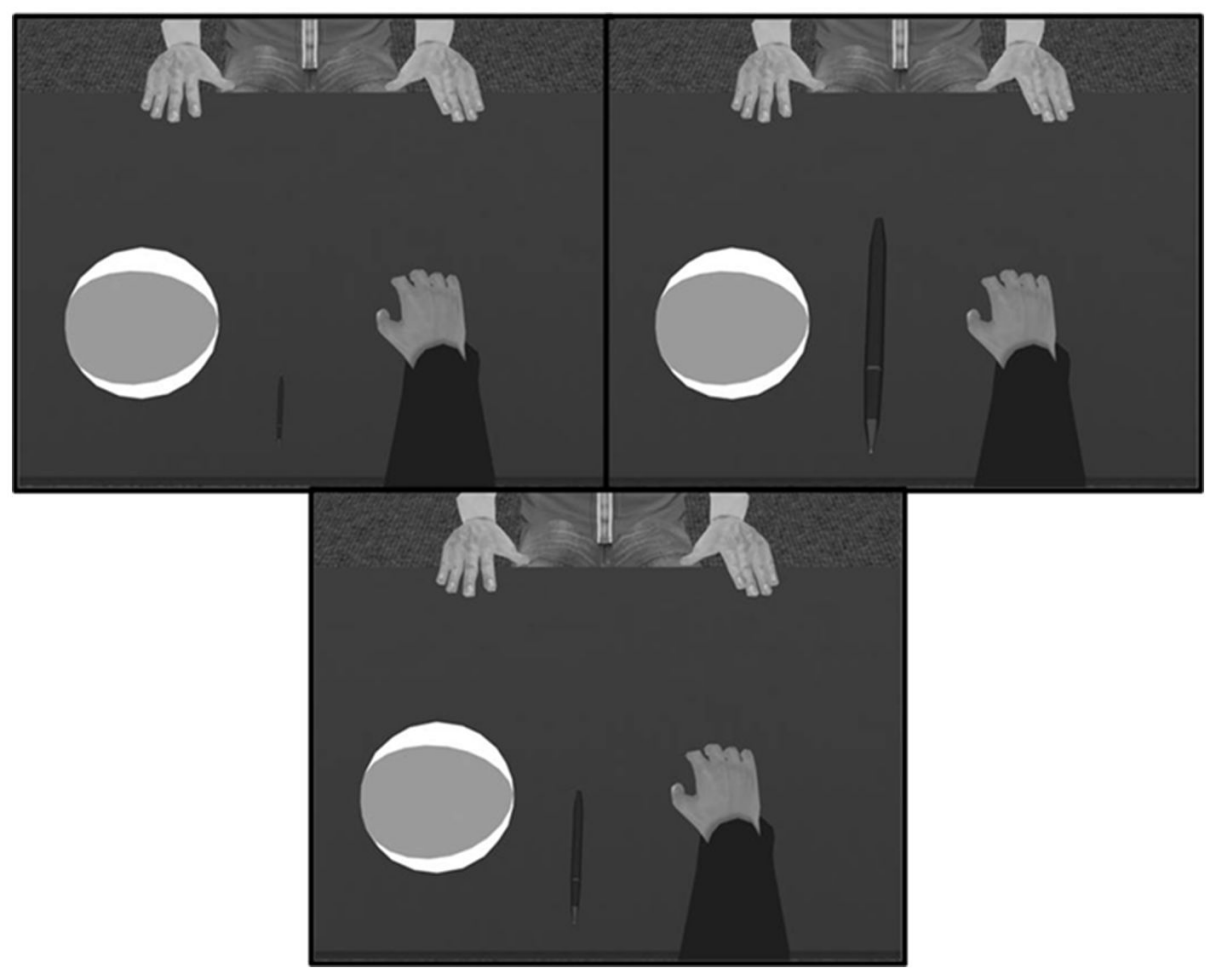
Experimental setup for Experiment 3 with respect to the participants’ viewpoints in the three different pen size conditions.

The procedure was the same as in Experiment 1; however, instead of manipulating the size of the participants’ virtual hand, virtual fountain pen was positioned on the table a few centimeters to the right of the balls, and its size was manipulated across blocks. The relative change in the pen’s size corresponded to the relative change of size of the virtual self-hand in Experiment 1. The sizes of these virtual pens were small (L: 9.5 cm, Diameter: 0.625cm), medium (L: 19 cm, Diameter: 1.25), or large (L: 38 cm, Diameter: 2.5cm), see Figure 6. Participants had a self-animated hand as in Experiment 1, except in all conditions, it was the medium sized hands.

## Results

Size estimates were analyzed using a repeated measures ANOVA with actual ball size and pen size as within-subjects variables and size estimate as the dependent variable. Actual ball size was significant with individuals giving larger estimates for larger balls, F(11,121)= 114.83, *p*< .01, *ŋ*_*p*_^2^ = .91. Pen size was not significant, *F*(2,22) = 2.58, *p*=.10, with individuals providing similar estimates in the large, *M*= 4.60*, SE*=.28, medium, *M*=4.81*, SE*=.41, and small pen, *M*=4.33*, SE*=.39, conditions.

These results show that another familiar object does not elicit similar results in size scaling as the observer’s own hand. As advocated in body-based perceptual scaling, these results suggest that one’s own body plays a privileged role in the perception of sizes. Similarly, these results provide evidence against a potential argument that these results are due to familiar size cues. If this were the case, then the pen should have elicited some effect on perceived size although smaller in magnitude than the hand as it is likely less familiar. However, the pattern of the null results in this study was inconsistent with what one would expect in the case of familiar size scaling. Additionally, the manipulation, methods, and instructions in this experiment were very similar to those in Experiment 1. If one was to attribute the results in Experiment 1 to demand characteristics, then one should also expect the same demand characteristics in this experiment. However, no effect was observed, which supports the notion that results in Experiment 1 are a result of body-based perceptual scaling rather than demand characteristics.

### Experiment 4: Hand Dimensions and Shape Perception

Although many studies rely on verbal reports as dependent measures of perceived extents and verbal reports are highly correlated with other measures of extent [24], it is also important to supplement these findings with less transparent measures of perceived size in order to control for possible response bias [25].

**Figure 7:**
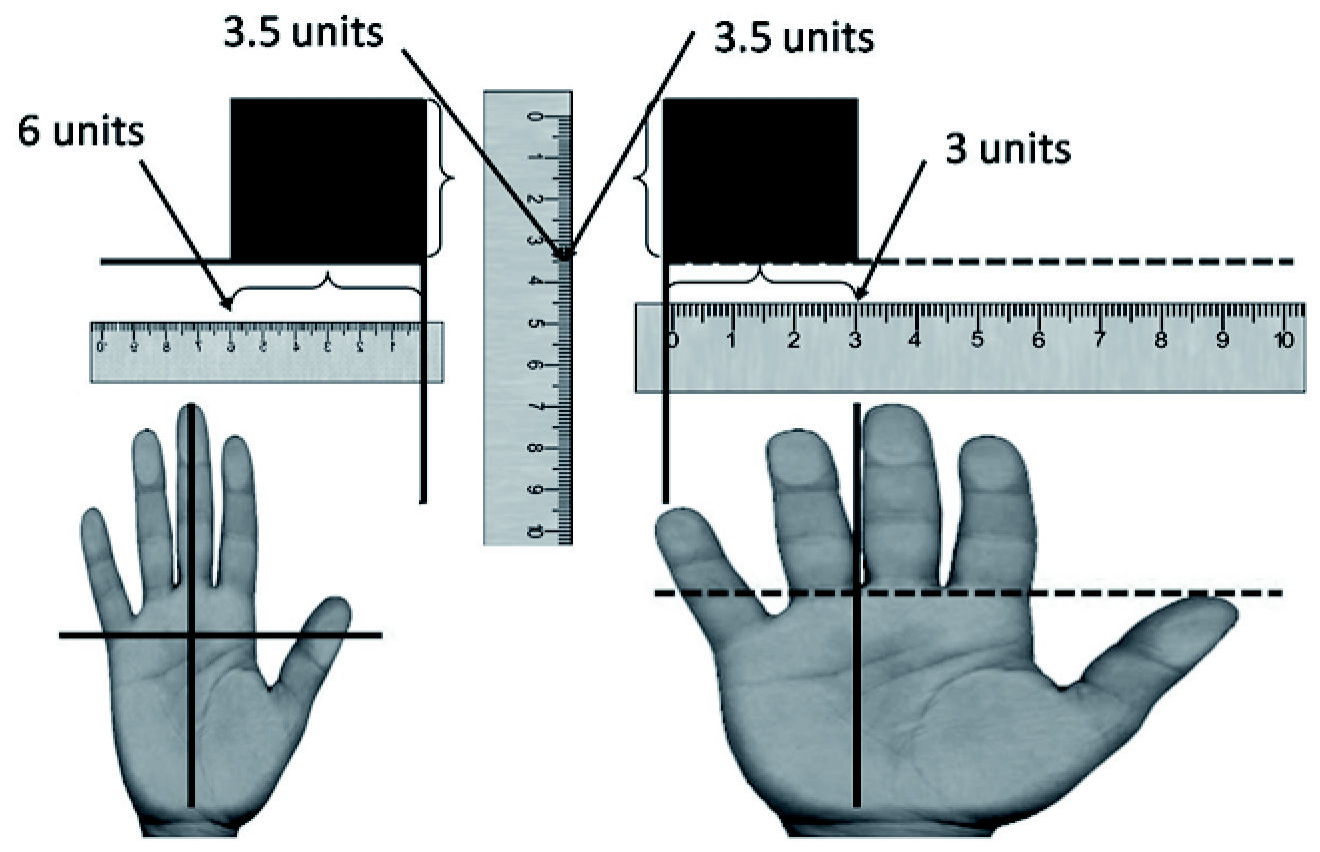
Illustration of body based scaling measurements across different hand width conditions.

Therefore, we investigated the influence of virtual body dimensions on the perception of the stimuli’s aspect ratio. We did this by manipulating only the width of the participant’s virtual hand, while leaving the length of the virtual hand the same. Participants were presented with a virtual box and told to estimate their ability to grasp the box across the width. Presumably, if the width of the boxes are perceptually scaled to the width of the hand and the length of the boxes are perceptually scaled to length of the hand, then the perceived relationship between the length and width of the boxes should vary as a result of changes in the width of the virtual hand. Put simply, when hand width is large, the width of the block should seem smaller relative to the length of the box than when hand width is small. As illustrated in Figure 7, if the length of the box is scaled to the length of the hand, then the perceived length of the boxes in both hand size cases should remain the same, in this case 3.5 “hand length units”. However, if the width of the hand is used to scale the width of the box, because the hand on the left has a smaller width than the hand on the right, the box width will be measured as smaller (6 versus 3 “hand” width units). Therefore, when the hand’s width is smaller, individuals should perceive the width of the boxes to be larger than the length more often than when the hand’s width is larger.

### Methods

#### Participants

Twelve (4 female) individuals participated in this experiment. Participants were recruited from the university community around Tübingen, Germany and were compensated for their time at a rate of eight € per hour. All participants started the experiment by completing a written consent form, which along with this study was approved by the ethical committee of the University of Tübingen, Germany for this study.

#### Stimuli & Apparatus

The experiment was conducted in a large, fully tracked walking space (4m x 6m). The same table and chair from Experiment 1 were placed in the center of the walking space. The table was covered with a black table cloth to decrease interference with the tracking technology. The position of participants’ hand and head were tracked using an optical tracking system (4 Vicon MX13 cameras, same as in Experiment 2). Participants wore the same tracking apparatus on their hand as in Experiment 1. Participants wore the nVisor SX111 head-mounted display (HMD) that displayed a stereoscopic image of the virtual world with a resolution of 1280x1024 pixels per eye, a latency of 40.8 ms and a FOV of 111 degrees diagonally. We decided to use this HMD due to its greater field of view than the HMDs used for Experiments 1 and 2.

**Figure 8:**
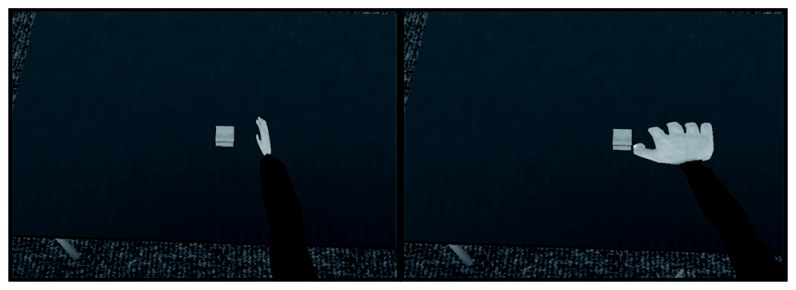
Screenshots of the different hand size conditions in Experiment 3.

The virtual environment was the same as was used in Experiment 1, except that the avatar sitting across the table from the participant was removed to allow for more space on the table due to the addition of a calibration phase which will be described below. For training, 4 dot pairs were created which consisted of two circles that where 2 cm in diameter and 1 cm thick; each circle had a checked grey and white texture. Dot pairs differed in the horizontal distance from the left to the right dot: 10, 12.5, 15, and 20 cm. The dot pairs where presented on the table either at the participant’s midline or 18 cm to the right and left of the midline. Dot pairs were presented at 5 different distances vertically from participants (as measured by the edge of the table). These distances were 3, 6.8, 10, 12.5 or 15 cm. Therefore, with 15 possible positions that each dot pair could be situated, there were a total of 60 possible dot pair-location combinations. For testing trials, 48 different virtual square boxes of various lengths and widths were created all with a height of 2 cm. All boxes had a wood grain texture, see Figure 8. Boxes could be one of 4 various widths: 10, 12.5, 15, or 20 cm. The lengths of the boxes varied from +/- 0, +/-1.5, +/- 3, +/- 4.5, +/- 6 mm from each width making a total of 12 boxes of different lengths for each box of a certain width (hence 4 widths x 12 lengths = 48 boxes).

#### Procedure

Participants sat at the table while wearing the HMD as well as the hand tracking apparatus. Participants completed two blocks of training and testing. In one block, the width of the virtual hand was increased to be 2x its normal size. In the other block, the virtual hand width was decreased to be 1/2 of its normal size. In both blocks, the length of the hand remained the same and was not manipulated, see Figures 7 and 8.

Each block began with a training phase to calibrate the participant to their virtual hand. A trial consisted of one dot pair being presented at one of the locations. The participants were instructed to touch the thumb of their virtual hand to the left dot in the dot pair and then touch the pinky of the virtual hand to the right dot in the dot pair. After participants touched both dots in the dot pair, the dot pair disappeared and another dot pair appeared. Participants were presented with all 60 dot pairs in random order. There were no time constraints and participants were allowed to take as much time as they needed to complete training trial.

After participants completed the training phase, they immediately began the test phase. In the test phase, participants were presented with one of the boxes. Participants were instructed that they were to make an estimate of whether they could grasp the boxes in the virtual environment with their virtual hand. For this grasping ability estimate, they were instructed that although the virtual fingers were not animated they should estimate their grasping ability with respect to what the hand could accomplish if the fingers were animated. They were also told that they were only to estimate whether they could grasp the box across its width; they were told not to consider grasping the boxes in any other way. Participants were then instructed to respond as to whether the length or the width of the box appeared larger. Each box was presented until the participant made both the grasping ability and aspect ratio response. After participants made both responses, the current box disappeared and was immediately replaced with a new box and the next trial began. In each block, each box was presented twice for a total of 96 trials per block. Participants completed two blocks; one in which the width of the virtual hand was doubled and the other in which the virtual hand was halved. The length of the hand remained the same across different blocks. Block order was counter-balanced across participants.

## Results

To ensure that there was an actual difference in perceived grasping ability across hand width conditions, we calculated the ratio of boxes graspable by dividing the number of boxes graspable by the total number of boxes per participant and per hand width condition. Using a repeated measures ANOVA with hand width as the independent variable and ratios of boxes graspable as the dependent measure, we found that participants estimated more boxes as graspable when their virtual hand width was larger, *M* = .90, *SE*=.03, than when their virtual hand width was smaller, *M* = .61, *SE*=.08, *F*(1,11) = 18.36, *p*< .01, *ŋ*_*p*_^2^ = .63. Therefore, we can confirm that the manipulation of hand width influenced perceived grasping ability.

**Figure 9:**
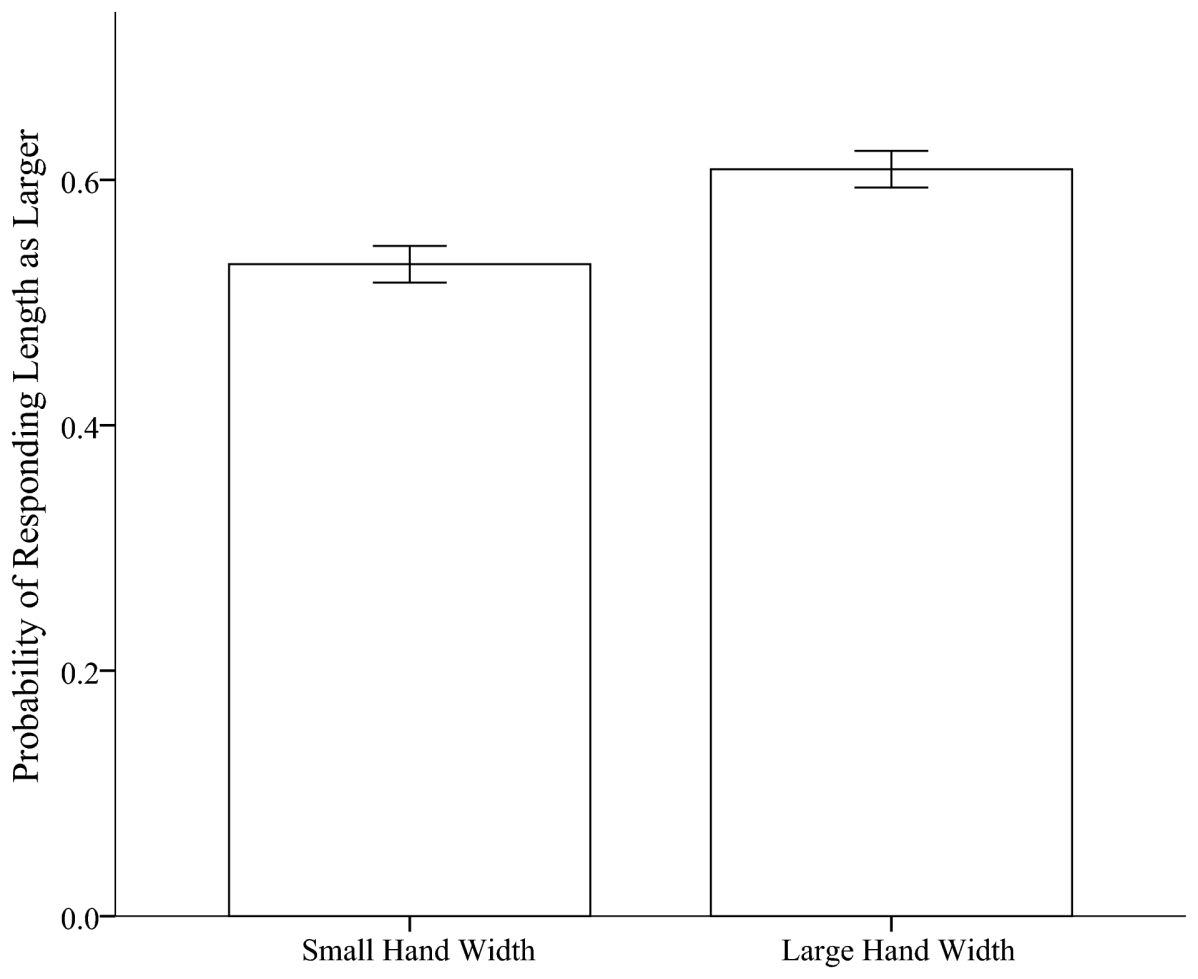
Percent of responding length is larger in both hand width conditions. Error bars represent 1 standard error and are calculated on the basis of within-participant error with the method provided by Loftus and Masson [37].

To assess whether hand width influenced the perception of the dimension of the boxes, we calculated the ratio of length responses for each participant for each hand width condition by dividing the number of length responses by the total number of dimension responses. Recall the hypothesis, that if hand width influenced the relevant aspect of object width and not the irrelevant aspect of object length, then when hand width is larger, the object width should be scaled as smaller. When hand width is smaller, the object width should be scaled as larger. As a result, if hand width influences object width but not length, the individuals which experience the wider hand should respond the length is longer more often than those who experience a shorter hand width. As assessed by a repeated measures ANOVA with ratio of length responses as the dependent variable and hand width and the difference between box length and width as an independent variables, we found that participants responded that the length was larger significantly more when hand width was larger, *M* = .61, *SE*=.04, than when hand width was small, *M* = .53, *SE*=.04, *F*(1,11) = 7.02, *p*= .02, *ŋ*_*p*_^2^ = .41, see Figure 9. As can be seen, individuals responded that the length was larger more often than the width; however, this can be explained through the well known phenomenon that individuals typically underestimate the horizontal with respect to the vertical [26].

**Figure 10:**
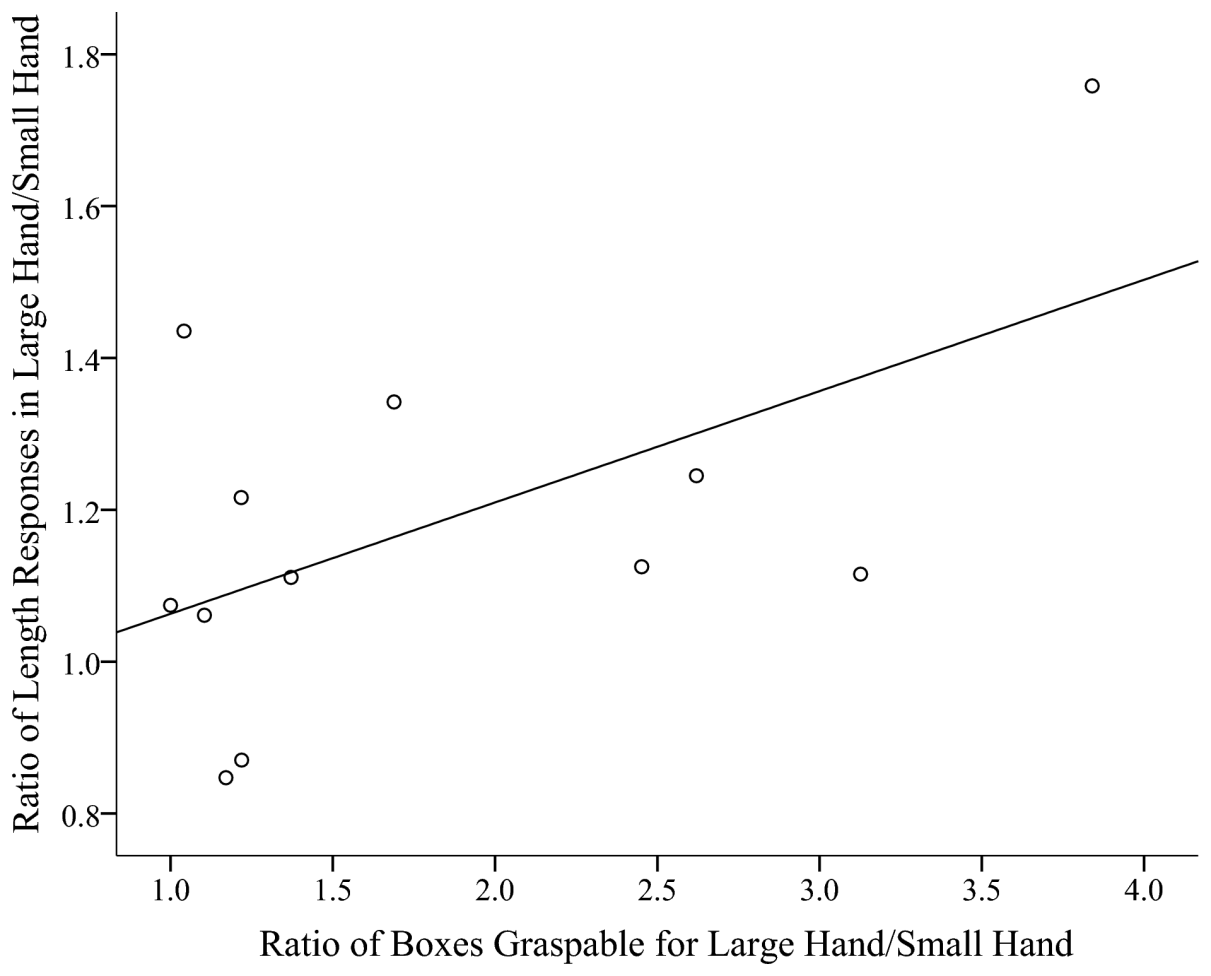
Change in proportion of perceived length responses between large and small hand width condition as a function of the proportional change in perceived grasping ability between the large and small hand width conditions. Each circle represents one participant’s data, and the solid line is the correlation between the two plotted variables.

Additionally, we investigate whether there is a relationship between change in perceived object dimension and change in perceived grasping ability across hand width conditions. In order to do this, the ratio of boxes graspable in the large hand width condition was divided by the ratio of boxes in the small hand condition. This creates a change in grasping ability variable that relates the proportional difference in grasping ability from the large hand width condition from the small hand width condition. Similarly, the ratio of length responses in the large hand width condition was divided by the ratio of length responses in the small hand width condition. This creates a change in the dimension variable that communicates the percent increase in length responses in the big hand width condition over the small hand width condition. As hypothesized, we found a positive correlation between change in perceived dimension and change in perceived graspability, *r* = .56, *p* = .03, one-tailed test, see Figure 10. This correlation strengthens the assertion that perceived sizes are scaled to the relevant aspects of the hand. Additionally, this study shows these effects in a less transparent measure which supports previous findings using verbal reports.

## Discussion

This set of studies provides support for the notion that the hand acts as a metric which individuals use to scale the apparent sizes of objects in their environment. Similarly, these studies highlight not only the importance of the visual (in this case virtual) body, but its size and dimensions in the perception of spatial layout in virtual environments. The first experiment showed increases in the perceived sizes of objects as a function of decreases in the size of one’s virtual hand. The second experiment showed that these results were not due to size contrast effects and that these effects only occur after changes in the size of one’s own body, not just any body part in the environment. The third experiment using differently sized pens confirmed that the results in Experiment 1 were not due to the virtual hand being the only familiar size cue in the virtual environment. The final experiment reinforced the hypothesis that people scale object size to the hand by showing the effect using a less transparent measure of perceived size, in this case, the perceived dimensions of stimuli’s aspect ratio.

These studies can be included in a growing body of research showing influences of the body and its action capabilities on the perceptions of spatial layout [3]. Presumably, these influences are a result of using the body and its action capabilities as “perceptual ruler” to which perceived sizes and distances are scaled. Optical information underlying the perception of spatial layout comes to the eye in the form of visual angles and changes in those angles. In order to perceive the dimensions of the spatial layout, this angular information must be rescaled to a metric that can be applied to distances and sizes. Until recently, this perceptual metric has not been extensively researched and direct hypotheses had been made about what this metric could be have not been tested. These findings and others suggest that the perceptual metric is derived from the body.

Because the meaning of an extent is derived from the metric to which it is scaled, interpretations of the purpose in having a perception of spatial layout are inherent in hypotheses about the source of these perceptual metrics. In several approaches which espouse the modularity of the visual perceptual system from other cognitive and motor processes, the purpose of perception is assumed to be the creation of a geometrically veridical 3-dimensional representation of external world [27,28] which would presumably be scaled to a arbitrary metric that is consistent across all sizes and distances in order to maintain geometric fidelity. However, from a biological and/or evolutionary perspective, one could assert that perceiving the spatial layout has everything to do with allowing the organism to effectively interact within its ecological niche irregardless of the geometric accuracy of the spatial perceptions. Indeed, several simulations of various perceptual representations have shown that even the most geometrically accurate are not the most evolutionarily effective [29]. As a result, these perceptual metrics would be derived from what was relevant to the perceiver with respect to their environmental actions. Arguably, there is nothing more relevant to environmental interaction than one’s body. The findings in these studies add to the body of research demonstrating that the relevant aspect of the body is used as a perceptual metric, which additionally supports this latter purpose. Put simply, the perception of spatial layout expresses the relationship between one’s action capabilities and one’s ecological niche instead of geometric consistency.

More specifically, in these studies, controlled manipulations of the size and dimensions of the hand produced changes in perceived size and shape in the directions predicted by body-based perceptual scaling. Although previous studies have found influences of hand size on object size perception, these studies were limited due to distorting the size of the body using lenses of fixed optical distortion or pre-existing differences. These limitations made it difficult to change the size of the hand in a precise manner or manipulate its dimensions. In the present set of studies, virtual reality and self-representing avatars have freed us from these limitations to determine how such changes influence size. The use of virtual environments and “virtual bodies” allowed us to manipulate aspects of the body and the environment that would be impossible to manipulate in the real world. We were able to make specific and precisely controlled changes in the “body” of the perceiver to assess its impact on the perception of spatial layout.

One alternative explanation for the results in the first experiment was that the findings were due to size contrast effects, i.e. objects appear to be smaller when surrounded by larger objects and vice versa. Therefore, Experiment 2 sought to control for size contrast effects by providing a non-self representing virtual character whose hands positioned on the table beside the target objects were manipulated in size. This design also allowed us to investigate whether this size scaling can occur via a body aside from one’s own. Previous findings associated with body-based perceptual scaling have implied that these changes must be associated with one’s own body rather than another’s body [30]. Objects were perceived as the same size regardless of the virtual character’s hand size in Experiment 2, which shows that it is unlikely that size contrast effects can explain the findings in Experiment 1 and also that one’s own body is important when scaling perceived object size. However, it is important to note that individuals’ hand motions did not animate the avatar’s hands as they did their own virtual hands in Experiment 1. So, it is possible that given self-animation in which the virtual character’s hands are functionally relevant to the perceiver that they would scale objects sizes to the hands. Similarly, research on motor simulation has provided ample evidence that individuals utilize their own motor systems when viewing the actions of others (see 31, for a review), and other research has implicated motor simulation as a possible mechanism for body-based perceptual scaling [32]. As a result, it is possible that if the participant was capable of performing actions with the 3^rd^ person virtual character’s hands (animated movements of arms and hands), then individuals may have scaled the sizes of the objects to the avatars hands due to motor simulation. These possibilities will be an interesting direction for future research.

Familiar size as a depth cue is another potential interpretation of the change in perceived object size in these studies. Familiar size is a well established size cue that can be described as the use of the familiar size of known objects to ascertain the unknown sizes of unfamiliar objects in the same visual field [33]. It could be proposed that body-based scaling is merely a special case of using familiar size cues. We would not disagree with that assessment, but rather extend that view to more clearly define the basis for which an object’s size becomes familiar. The notion of a familiar size can be recharacterized as the awareness of the relationship between the size of the body and the size of an object. After all, knowing the size of an object must be anchored to some relative metric, and the body is really the only relevant thing we have to which sizes can be compared with respect to an evolutionary perspective (consider the big and little Alice example from the introduction). Thereby, using familiar size as a depth cue could be described as using the known relationship between the body and an object to ascertain the size of another object. Put simply, we would suggest that familiar size scaling is likely a form of indirect body-scaling. Our results support this notion as they show that, especially through Experiments 2 and 3, that even in the event that familiar objects are available, one’s own body trumps all. However, this is a speculative interpretation, and more research would be required to investigate the merit of this claim.

Several criticisms of body-based perceptual scaling have argued that size and distance perception are not influenced by the body and that effects are due to response biases and/or demand characteristics [34,35]. We conducted Experiments 2, 3 and 4 to address the concerns of possible response bias or demand characteristics influencing our experimental results. Given the null results in Experiments 2 and 3, there is no plausible explanation as to why participants in Experiment 1 would exhibit demand characteristics and other participants in Experiments 2 and 3 would resist exhibiting demand characteristics. The manipulations in Experiments 2 and 3 were extremely similar to the manipulations in Experiment 1 down to manipulating the size of a perceptually similar hand (Experiment 2) or familiar object used with the hand (Experiment 3) by the same scaling factor. However, one could argue that response bias is influenced by cue familiarity and that one’s own hands were the most familiar manipulation. However, the non-significant and slight mean differences in alternative directions in Experiments 2 and 3 argue against this postulation as one would expect at least some small effect in the same direction as Experiment 1 in these control experiments.

Our analyses showed that in Experiment 1 differences in the magnitude of the effect of hand size increased as a function of ball size, and no difference was present at the y-intercept as would be predicted by a scaling difference account. Response bias is typically characterized by a consistent shift in responses across all estimates resulting in an intercept difference rather than a slope difference. We find it unlikely that participants predicted that they should vary their compliance to experimenter demands contingent on the ball size which they were estimating. Finally, we conducted Experiment 4 with a psychophysical design, which is less susceptible to demand characteristics, with a design in which the participants were highly unlikely to deduce the hypothesis, before interpreting our results of Experiment 1 in support of body-based scaling. Drawing from these points, it is highly unlikely that the results from Experiments 1 and 4 are due to demand characteristics or response bias.

Additionally, individual differences as are less subject to demand characteristics and response bias, because the hypotheses are difficult or even impossible for participants to anticipate because there is no manipulation. These alternative methods can be used in order to assess perceived distance and size. Indeed, many studies have shown that these body-based perceptual scaling effects can be shown by using indirect measures of perceived extents or by taking advantage of individual differences in which no manipulation is necessary [4,6,13,36]. In Experiment 4, we showed not only the influence of hand width on an less transparent measure of size, but we also show that individual differences in individuals perceived action capabilities influenced the magnitude of these effects. When participants hand width was larger, they reported that box length was larger more often than when their hand width was smaller. Presumably, when they were required to estimate their grasping ability across the width of the box, participants scaled the width of the box to the width of their hand. When their hand width was large, the width of the box appeared smaller in comparison to the length. When their hand width was smaller, the width of the box appeared larger in comparison to the length. Similarly, the magnitude of this effect increased as a function of the change in participants’ change in perceived grasping ability across the two hand width conditions. These effects are difficult to attribute to response bias or demand characteristics, because it is highly unlikely that individuals could surmise whether and how their responses of the dimensions of the box should vary with respect to the manipulation. Similarly, it is also highly unlikely that the individual differences found in the relationship between perceived grasping ability and the magnitude of the difference in shape perception between hand size conditions can be explained through response biases or demand characteristics. Therefore, the results in Experiment 4 provide convincing support that the results found in these studies are due to differences in body-based perceptual scaling.

Because these studies were run in virtual environments, one cannot be sure that these findings can generalize to real environments. Due to less depth cues and less realism (the hand they were looking at did not have the same realism or identity as their physical hand) it is possible that individuals could be influenced by information in the virtual environment that they would not normally be influenced by in real world environments. However, the virtual environment that individuals were tested in was a full-cue environment, full of objects of familiar size and perspective cues in addition to stereoscopic information and motion parallax. In other words, there were ample depth cues available in the environment to fully specify the object’s sizes. Importantly, we are not contending that the virtual hand size influenced optical information specifying depth, but rather it provided the metric that scales the information these depth cues provided. Importantly, these studies in VEs are also in accord with previous findings in the real environments. Enlarging the virtual hand produced a similar effect (albeit larger) on perceived object size as found in real environments when the hand was visually enlarged by placing it under a magnifying sheet or by attempting the grasp with the perceptually larger right hand [13,14]. It is also possible that individuals may interpret changes in their bodies differently in surreal environments than in real environments. However, measures of physiological and behavioral responses have shown that individuals respond to stimuli with their virtual bodies in similar ways in which they respond with their own physical body [15,18]. So although individuals may know the virtual world is not real, they seem in many cases to treat it as though it is real.

Although the optic variables that inform perceived size are well understood, the metric to which these variables are scaled has received little attention, and consequently, the perceptual metrics used in many situations is largely unknown. Because the body and its abilities define the action relevance of objects’ sizes in our environment, it stands to reason that some evidence supports the notion that certain aspects of the body can be used as perceptual metrics [2]. The studies presented in the current paper provide evidence that one’s virtual body can influence perceived object size in virtual environments as well as substantiate corresponding findings in the real world in which body size manipulations were less controlled. Larger virtual hand sizes led to decreases in perceived object sizes; however, only when the virtual hand was perceived in first-person and animated with the movements of the perceiver. Large virtual hands of a virtual character and a large familiar object did not influence perceived size. Finally, less transparent measures of perceived size were also influenced by the dimensions of the virtual hand which argues against an explanation associated with demand characteristics and response biases. These results can be interpreted with respect to a body-based, perceptual scaling approach in which perceivers scale the optical variables specifying the sizes of objects to the relevant aspects of their bodies. Additionally, these results highlight the need to consider the role of the body and its action capabilities in not only how spatial perceptual information is acquired, but also in the interpretation of spatial perception in general.
